# Xenon Diffusion Mechanism and Xenon Bubble Nucleation and Growth Behaviors in Molybdenum via Molecular Dynamics Simulations

**DOI:** 10.3390/ma12152354

**Published:** 2019-07-24

**Authors:** Wenhua Zhang, Di Yun, Wenbo Liu

**Affiliations:** 1 School of Nuclear Science and Technology, Xi’an Jiaotong University, Xi’an 710049, China; 2 State Key Laboratory of Multiphase Flow in Power Engineering, Xi’an Jiaotong University, Xi’an 710049, China

**Keywords:** Molecular dynamics, xenon bubbles, diffusion mechanism, nucleation

## Abstract

The behaviors of xenon in molybdenum were studied using molecular statics and molecular dynamics simulations. The diffusion mechanism of xenon atoms was studied combining molecular dynamics, nudged elastic band, and temperature-accelerated dynamics methods. The vacancy-assisted diffusion mechanism was analyzed and the corresponding energy barriers were calculated. The clustering process of scattered xenon atoms was studied at an elevated temperature. Xenon bubbles were observed to form when the concentration of xenon atoms exceeded a threshold concentration value. Meanwhile, the interaction of xenon bubble and vacancies was investigated via the nudged elastic band method. The results showed that there exists a region around the xenon bubble where the migration energy of vacancy is significantly influenced. This work provides useful insights towards a better understanding of the behaviors of xenon in molybdenum.

## 1. Introduction

A comprehensive understanding of the irradiation behaviors of nuclear fuel is of great importance to the safety of nuclear reactors. As one of the typical irradiation behaviors, fuel swelling due to fission gas atoms has gained widespread attention [1,2]. Due to their extremely low solubility in nuclear fuel materials, fission gas atoms such as Xe and Kr tend to precipitate into bubbles. The formation of gas bubbles not only affects the mechanical and thermal properties of the fuel but also enhances fuel swelling which may lead to mechanical stresses on cladding materials. Besides, fission gas species may be vented to the fuel plenum, increasing the plenum pressure. Excess fuel plenum pressure and/or fuel swelling as a result of gas bubble growth may eventually lead to fuel element failure. Severe fuel swelling may even disturb the coolant flow path leading to insufficient cooling. Therefore, a complete understanding of the fission gas bubble behaviors is necessary.

Many experimental and modeling studies have been performed in order to improve the understanding of fission gas behaviors and enhance the ability to predict fission gas release during reactor operation [3,4,5,6]. In-pile data is the most important source for investigating the gas bubble behaviors, but one of disadvantages of in-pile experiments is the lack of sufficient understanding of the whole physics process. Advanced experimental techniques such as in situ ion irradiation via transmission electron microscopy (TEM) [7] can capture the continuous growth of gas bubbles and obtain the number density and the size distribution of the gas bubbles. However, it is difficult to characterize the bubble nucleation process and the early growth stage of small bubbles due to the inherent resolution limit of the TEM equipment. To this end, molecular dynamics (MD) can be a powerful supplement.

Modeling methods such as rate theory [8], cluster dynamics [9], and phase-field [10] that can be used to predict fission gas bubble behaviors need many key input parameters. Preferably, these parameters are obtained by experiments. However, the uncertainty of some of these key parameters, such as the diffusion coefficient, is very large and can even reach orders of magnitude [11]. The excessive uncertainty of parameters makes it hard to determine the physical model and fission gas behavioral mechanism, which seriously hinders the development of the fission gas mechanistic model. In order to resolve these issues, some researchers have proposed the multiscale simulation approach [12]. The multiscale simulation approach combines the modeling methods from atomic scale to intermediate grain scale to fuel pellet/fuel rod continuum scale. MD is an important atomistic simulation tool based on empirical interatomic potential. It not only can help us elucidate the basic mechanisms of fission gas behaviors in nuclear fuel, but also can calculate the key parameters for mesoscopic scale simulation thus reducing the uncertainty of modeling. Therefore, MD plays an irreplaceable role in the study of fission gas behaviors.

Xenon (Xe) is the primary gaseous fission product and there are many studies using MD to simulate the behaviors of Xe bubbles in UO_2_, including the diffusion mechanism [13,14,15], nucleation of Xe bubbles [16,17,18,19], etc. However, due to the complexity of the UO_2_ fuel material (U and O sublattices, grain boundary effects, etc.) and the Xe bubble behaviors, it is difficult to pin down the fission gas behavioral mechanisms. In order to solve this problem, some separation effect experiments have been proposed, which attempt to simplify the complex material system and only characterize one or few physical processes of the fission gases in order to clarify the underlying behavioral mechanisms.

As one of such attempts, Di Yun et. al. [20] performed a Xe ion implantation experiment via in situ TEM in single-crystal molybdenum and characterized Xe bubble morphology and behaviors. The dynamic evolutions of Xe bubbles in the crystal were observed continuously and real-time, and some key information such as the size distribution of the Xe bubbles was obtained. This experiment attempted to provide useful insights for irradiation damage and gas bubble behaviors in Mo. By selecting high-purity single-crystal molybdenum, this experiment aimed at simplifying the system, eliminating the influence of grain boundaries, and as such elucidating the fundamental mechanisms behind the physically observed phenomena. A multiscale simulation calculation was carried out to help interpret the experimental findings. In this work, the diffusion mechanisms of Xe atoms in Mo, the nucleation and early growth behaviors of Xe bubbles are studied, and the results serve the purpose of supplying behavioral mechanism information for the higher length scale simulation models and providing relevant information to help interpret some of the key experimental findings from the in situ Xe ion implantation experiment.

## 2. Simulation Method

### 2.1. The Interatomic Potential

The accuracy of MD simulation strongly depends on the interatomic potentials function used to support the simulation. In this paper, a ternary embedded-atom method (EAM) potential for uranium–molybdenum–xenon system developed by Smirnova et al using a force-matching technique and a dataset of ab initio atomic forces [21] was used. The potential is proved suitable for simulation of compounds and pure elements in the ternary alloys system. The computed elastic constants, thermal expansion and the melting temperature of U, Mo, Xe are consistent with the experimental data. The pressure–volume isotherm of Xe is reproduced with good accuracy, which verified that the potential is qualified to simulate the excess pressure Xe bubbles. Meanwhile, the calculated vacancy and SIA formation energies in U and Mo agree well with the ab initio calculation results, which proved that the potential may be used to predict the defect behaviors. Xiao H. [22] and Hu S. [23] also used this potential in their simulations. Consequently, it is believed that the potential is suitable to be used for our simulation.

### 2.2. The MD Simulation Setup

A MD simulation program LAMMPS [24] was employed for all the simulations in this work. For all the MD simulations, the time step is set as 0.001 ps. The temperature is controlled via a Nose/Hoover temperature thermostat and the periodic boundary condition is used. For static relaxation, the energy minimization is performed and the minimization algorithm is set as the conjugate gradient method (cg), with the stopping criteria for energy tolerance of 10e−12 s^−1^ and the force tolerance of 10e–12 eV/Å. The specific simulation process for different behaviors is different and the details of different simulation are described respectively as follows.

#### 2.2.1. Stable Configuration of Xe in Mo

In order to confirm the stable site of Xe in Mo, the lowest energy configurations of the $Xe_{n}V_{m}$ cluster were obtained by randomly putting m Xe atoms into a vacancy cluster and performing molecular static (MS) simulation for each configuration. MD simulation was performed after generating each configuration to equilibrate the system. The simulation system containing $Xe_{n}V_{m}$ clusters was ramped up to 500 K and then relaxed for 10 ps followed by cooling down to 0.0001 K.

#### 2.2.2. The Diffusion Mechanism of Xe in Mo

The effect of a substitutional Xe on the nearby vacancies in Mo was studied by MD and the Nudged Elastic Band (NEB) method [25,26,27]. In the MD simulation, the size of the simulation system was set as 20 × 20 × 20. The corresponding vacancies and substitutional Xe atoms were created in the perfect single-crystal molybdenum system. Then the temperature of the system was raised to 500 K for 100 ps and cooled to 0.0001 K for 5 ps in the NVT ensemble for full relaxation. The size of the simulation system in the NEB calculation is the same as that in the MD simulation.

Then NEB and temperature-accelerated dynamics (TAD) [28] methods were used to study the vacancy-assisted diffusion mechanism. In the NEB simulation, the size of the system was set as 20 × 20 × 20. While in the TAD simulation, the size of the system was set as 10 × 10 × 10. The high temperature and low temperature used in the TAD simulation was 2400 K and 2000 K, respectively. The migration of Xe atoms was probed every 0.1 ps. If the Xe atom has moved a distance greater than 1.6 Å, it is considered that the Xe atom has migrated. The minimum pre-exponential coefficient set in the TAD simulation was 10e−12 s^−1^, and the desired confidence level is 90%.

#### 2.2.3. The Nucleation of Xe Bubbles in Mo

MD simulation was performed to simulate the nucleation process of Xe bubbles. The specific simulation process is as follows.

1. Construction and relaxation of the initial system: a bcc molybdenum system with 2% substitutional Xe atoms of 50 × 50 × 50 in size was constructed. The system was relaxed in the NPT ensemble at temperature of 300 K and zero pressure.

2. Relaxation of the above configuration with high temperature: after the equilibrium configuration at room temperature was obtained, the temperature was raised to 2500 K to accelerate the diffusion of Xe atoms. The system was relaxed in the NPT ensemble with zero pressure.

3. Evolution of the system: then a simulation was carried out in the NVT ensemble at 2500 K for 2 ns.

#### 2.2.4. The Influence of Xe Bubbles on the Surrounding Vacancies

When studying the interaction between Xe bubbles and the nearby vacancies, the migration energy of a vacancy around Xe bubbles were calculated using the NEB [27] method as implemented in LAMMPS. The specific simulation process is as follows:

1. Construction and relaxation of Mo system containing a Xe bubble: a molybdenum system of 50 × 50 × 50 were constructed and a Xe bubble of radius of 1 nm were created by removing all the molybdenum atoms in the region and adding Xe atoms. In order to fully relax the system, the system was heated to 500 K for 50 ps and cooled to 0.0001 K for 50 ps in the NPT ensemble at zero pressure. An equilibrium configuration containing a Xe bubble was obtained and the potential energy of each atom was calculated.

2. NEB was used to calculate the migration energy. The energy barrier of vacancy jumping towards the bubble at different distances around the bubble were calculated.

## 3. Results

### 3.1. Stable Configuration of Xe in Mo

**Figure 1:**
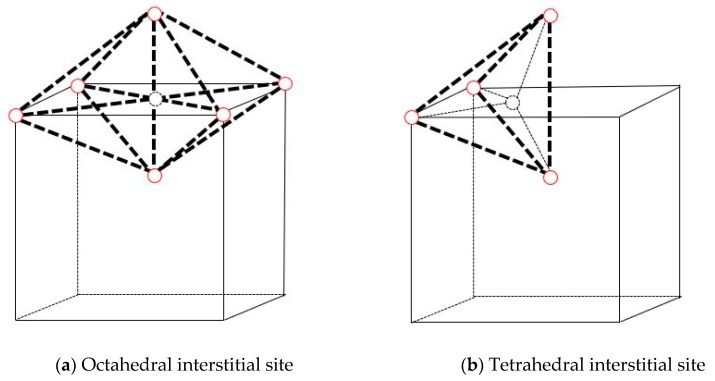
Interstitial site in bcc Mo (The solid red circles represent the molybdenum atoms and the dotted circle represents the interstitial site).

The formation energies of interstitial (on octahedral site and on tetrahedral site, shown in Figure 1) Xe and substitutional Xe atoms in Mo calculated in this paper and by the Density Functional Theory [29] (DFT) are shown in Table 1. These two results are in good agreement, which further provides the rationale of using the Smirnova potential. It can be seen that the formation energy of substitutional Xe is the lowest, and the formation energy of substitutional Xe + self-interstitial atom (SIA) is lower than that of an interstitial Xe. Therefore, it is energetically favorable for an interstitial Xe to displace a host Mo atom and become a substitutional Xe, producing an SIA at the same time, which is also observed in the dynamic simulations.

**Figure 2:**
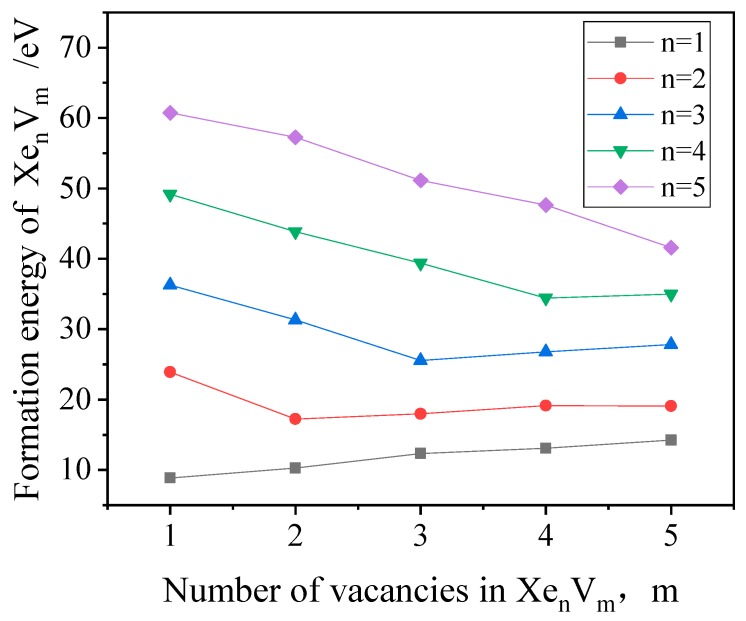
The $Xe_{n}V_{m}$ formation energy as a function of the number of vacancies (n and m are the number of Xe atoms and vacancies in the $Xe_{n}V_{m}$ clusters, respectively).

The formation energies of $Xe_{n}V_{m}$ clusters were also investigated in this study, where n and m are the numbers of Xe atoms and vacancies in the $Xe_{n}V_{m}$ clusters, respectively. The formation energy [30] of a $Xe_{n}V_{m}$ cluster can be calculated by the following Equation (1).

(1) $$E_{F}\left(Xe_{n}V_{m}\right)=E_{tot}\left(Xe_{n}V_{m}\right)-\left[nE_{Xe}^{c}+\left(N-m\right)E_{Mo}^{c}\right]$$

where N is the total number of Mo atoms in the perfect bcc crystal, $E_{Mo}^{c}$ is the cohesive energy of a single Mo atom in the perfect bcc Mo crystal, and $E_{Xe}^{c}$ is the energy of the isolated Xe atom. The calculated formation energies of $Xe_{n}V_{m}$, as a function of the number of vacancies, are shown in Figure 2. It can be observed that when the Xe-to-vacancy ratio is equal to 1, the formation energy of the $Xe_{n}V_{m}$ cluster is the lowest, which indicates that the lowest energy configuration occurs when the Xe-to-vacancy ratio is 1. Consequently, it is concluded that Xe is stable as a substitutional defect in bcc Mo, which is the same as in bcc U [31].

The binding energies can be used to estimate the stability of the $Xe_{n}V_{m}$ clusters. The binding energies of a Xe atom and a vacancy to the $Xe_{n}V_{m}$ cluster can be obtained using the formation energy of the $Xe_{n}V_{m}$ cluster that has been calculated above. The binding energy of a Xe atom to the $Xe_{n}V_{m}$ cluster is defined by the following Equation (2).

(2) $$E_{B}\left(Xe\right)=E_{F}\left(Xe\right)+E_{F}\left(Xe_{n-1}V_{m}\right)-E_{F}\left(Xe_{n}V_{m}\right)$$

The binding energy of a vacancy to the $Xe_{n}V_{m}$ cluster is defined by the following Equation (3).

(3) $$E_{B}\left(V\right)=E_{F}\left(V\right)+E_{F}\left(Xe_{n}V_{m-1}\right)-E_{F}\left(Xe_{n}V_{m}\right)$$

where $E_{F}\left(Xe\right)$ and $E_{F}\left(V\right)$ are the formation energies of an interstitial Xe and an isolated vacancy, respectively.

**Figure 3:**
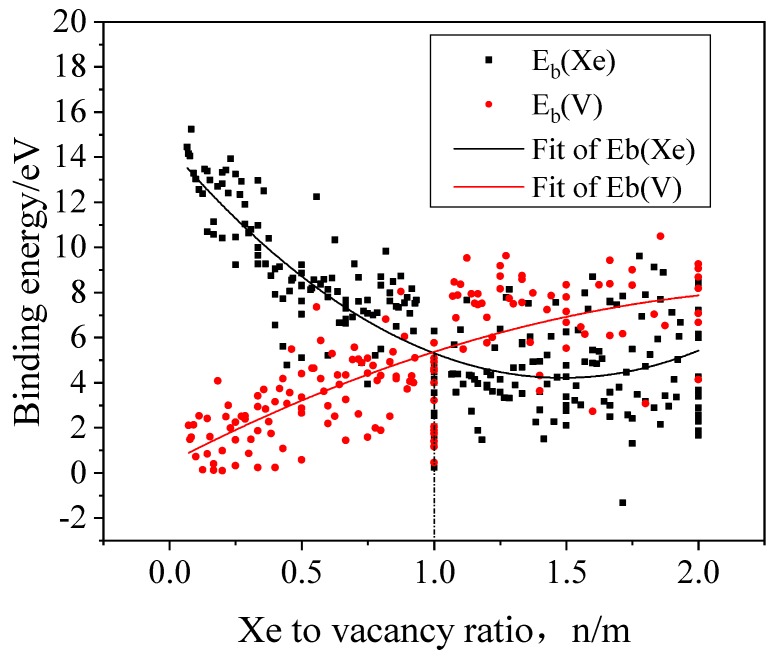
The binding energies of a Xe atom and a vacancy to a $Xe_{n}V_{m}$ cluster in Mo as a function of Xe-to-vacancy ratio.

In Figure 3, the calculated binding energies are shown as a function of the Xe-to-vacancy ratio, which displays a strong dependence of the binding energies on the Xe-to-vacancy ratio of the cluster. The higher binding energy of a Xe atom to the $Xe_{n}V_{m}$ cluster means stronger combination of the Xe atom and the cluster. Accordingly, the high vacancy binding energy suggests that it is energetically difficult to remove a vacancy from the $Xe_{n}V_{m}$ cluster. With the rising Xe-to-vacancy ratio, the binding energy of a Xe atom to the $Xe_{n}V_{m}$ cluster gradually decreases and the binding energy of a vacancy to the $Xe_{n}V_{m}$ cluster increases. When the Xe-to-vacancy ratio is less than 1, the Xe atom binding energy is larger than the vacancy binding energy, and as a result, the cluster is likely to combine with Xe atoms and increase the ratio. When the Xe-to-vacancy ratio is larger than 1, the binding energy of a vacancy to the $Xe_{n}V_{m}$ cluster is larger than that of a Xe atom, thus the cluster will tend to capture vacancies and decrease the ratio. Consequently, it becomes highly likely that the Xe-to-vacancy ratio is 1:1 for Xe bubbles in Mo from the view point of thermodynamics, though the Xe-to-vacancy ratio is also affected by the kinetics. Meanwhile, the binding energy of a Xe atom to the cluster is always larger than zero, which indicates that it is energetically favorable for Xe bubbles to absorb Xe atoms and grow larger.

### 3.2. The Diffusion Mechanism of Xe in Mo

After determining the stable site of Xe in Mo, one further step to study the diffusion mechanism of Xe is taken combining the MD, NEB, and TAD methods.

**Figure 4:**
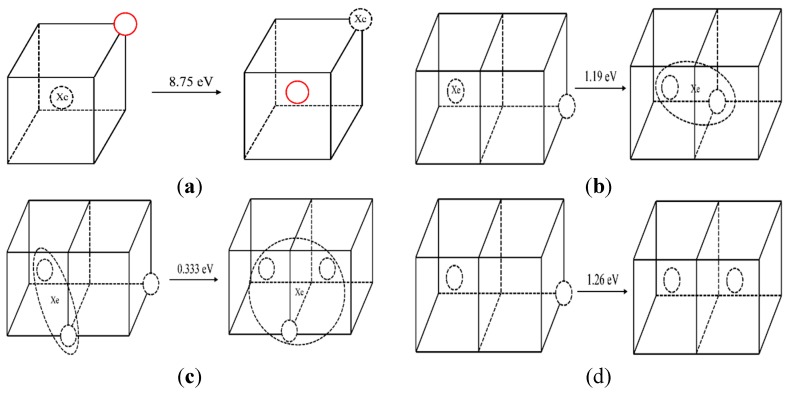
Diagram before and after a vacancy migration. (The solid red circle represents the molybdenum atom, the dotted circle represents vacancy, and Xe represents the Xe atom, the same below.)

The energy barrier of direct atoms exchange between a substitutional Xe atom and the nearest molybdenum atom is calculated by NEB. The atoms exchange schematic diagram is shown in Figure 4a. The solid red circle represents the molybdenum atom, the dotted circle represents vacancy, and Xe represents the Xe atom. The calculated energy barrier of a direct exchange is 8.75 eV. With such a high energy barrier, it is difficult for Xe to diffuse via this mechanism even at elevated temperatures. Thus, a vacancy-assisted diffusion mechanism of Xe is more likely.

In order to analyze the vacancy-assisted diffusion mechanism, the effect of a substitutional Xe on the nearby vacancies in Mo were studied by MD and NEB at first. Figure 4b,c shows the Xe–vacancy clustering processes when a vacancy was created at the fourth nearest site of the XeV or the XeV_2_ (Xe represents Xe atom and V represents vacancy) cluster. When a vacancy exists at the fourth nearest site of the Xe atom, the vacancy jumps to the nearest neighbor position of the Xe atom in the MD simulation. At the same time, the Xe atom relaxes to the middle position of the vacancies spontaneously. The corresponding migration energies of these two processes are 1.19 eV and 0.33 eV, as calculated by NEB.

The corresponding situations without Xe atoms in the system were simulated for comparison. In the NEB simulation, the migration energies of vacancy in these two processes without Xe atoms were 1.23 eV and 1.24 eV, respectively. In the MD simulation, when a vacancy appears at the fourth nearest site of a vacancy, the vacancy jumps to the second nearest position, as shown in Figure 4d. When a vacancy appears at the fourth nearest position of two vacancies, the vacancy is not absorbed by the divacancy cluster, but diffuses randomly in the matrix. At the same time, the calculated migration energy of a vacancy is 1.404 eV in single-crystal molybdenum which is close to D.G. Sangiovanni’s simulation result of 1.29 eV by DFT [32].

The migration energies calculated by NEB and the results of the MD simulation demonstrated that the substitutional Xe atoms in Mo contributes to the clustering of vacancies in Mo, thus forming a cluster of a Xe atom with two or three vacancies. The underlying driving force behind such agglomeration may be the stress field around the substitutional Xe atom.

Then NEB and TAD methods were used to study the vacancy-assisted diffusion mechanism from the perspective of energy and dynamics, respectively. It was observed that one of the vacancies in the clusters must migrate in order for a Xe atom to diffuse. Therefore, all possible configurations after one jump of a vacancy were studied. The different configurations are distinguished by the distance between vacancies and the Xe atom after one vacancy jump. The corresponding forward and reverse energy barriers were also calculated via NEB.

**Figure 5:**
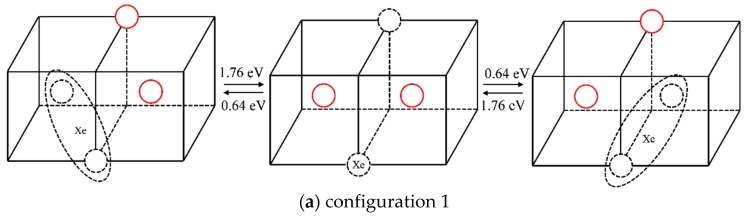

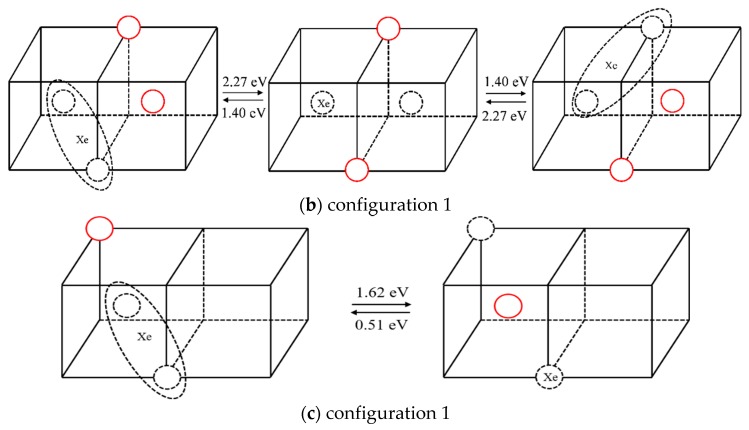
Different configurations and the corresponding migration energy barriers after one vacancy diffusion in XeV_2_.

**Figure 6:**
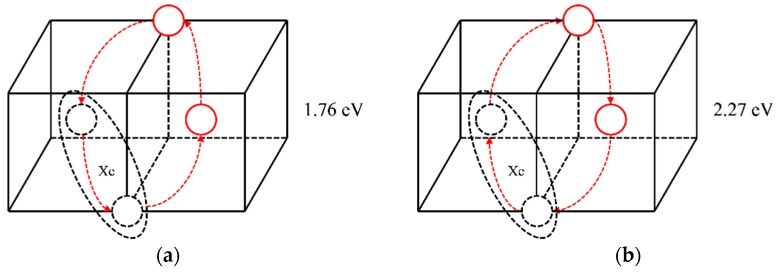
Synergic movement diffusion mechanisms of XeV_2_ and the corresponding migration energy barriers.

The statistical results for a Xe atom that combines with two vacancies (thus forming a XeV_2_ cluster) are shown in Table 2. The corresponding migration schematic diagrams are shown in Figure 5. The solid red circles represent the migrating molybdenum atoms and the dotted circles represent vacancies. For configurations 1 and 2, as shown in Figure 5a,b, the vacancy can jump to another nearest site of the substitutional Xe atom with one jump, and the Xe atom will relax to the middle of the two vacancies by migration. At the same time, in TAD simulation, the cooperative diffusion mechanism of a Xe atom, two vacancies and two matrix molybdenum atoms is observed, as shown in Figure 6. The corresponding motion pictures (Xe migration mechanism 1, 2.gif) are also provided as Supplemental Materials. For configuration 3, as shown in Figure 5c, it is impossible for the vacancy, after one jump to the upper left corner, to jump to the nearest site of the Xe atom with one jump except for jumping back to the original position. And the reverse migration barrier of the vacancy is relatively low (0.51 eV). It is therefore believed that Xe atom cannot migrate according to this scenario.

Based on the above analysis, it is shown that when a Xe atom combines with two vacancies, the synergic movement of the Xe atom, vacancy, and molybdenum atoms can lead to Xe migration. Both diffusion mechanisms shown in Figure 6 could occur. But the migration energy of the first diffusion mechanism shown in Figure 6a is lower, and it is therefore considered that this mechanism is more likely to occur.

**Figure 7:**
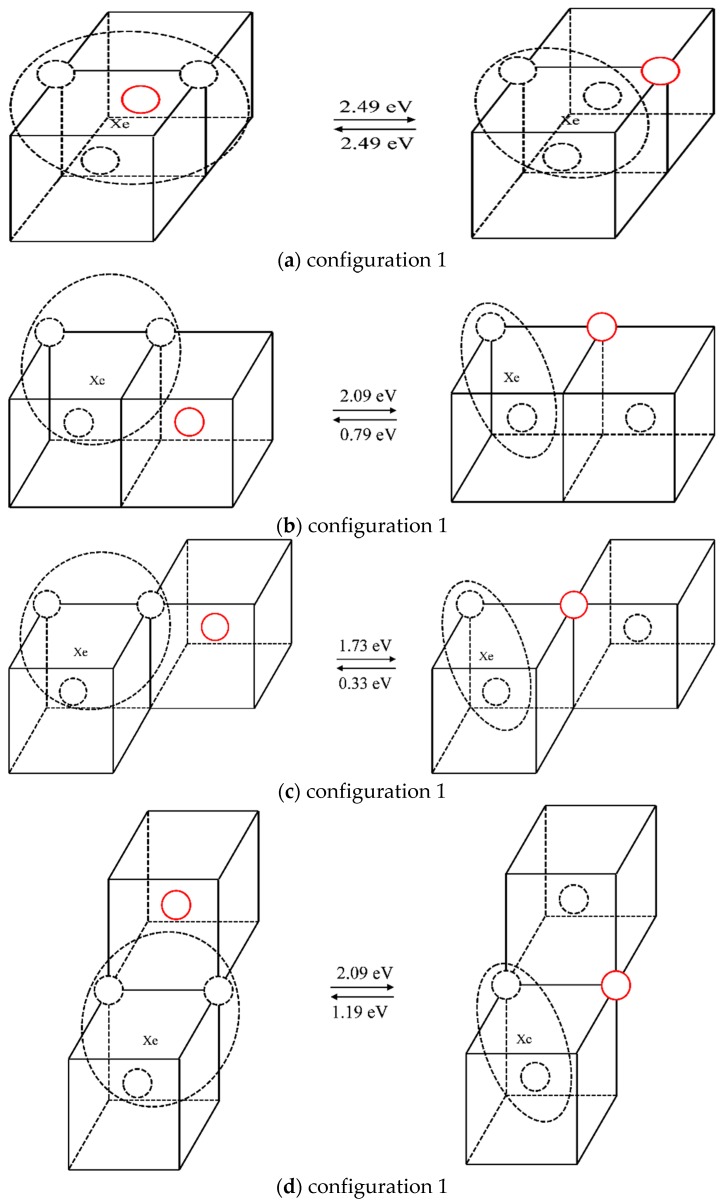

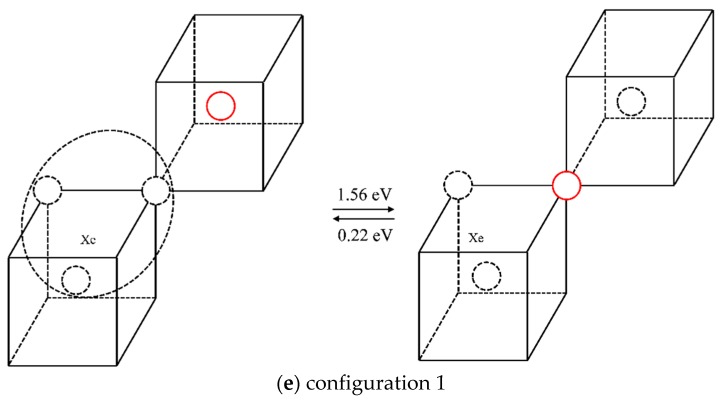
Different configurations and corresponding migration barriers after one vacancy diffusion step in XeV_3_.

The case of one Xe atom combined with three vacancies was studied in the same way as that used to study the scenario of one Xe atom combined with two vacancies. The different configurations, and the forward and reverse migration barriers of one vacancy jump are listed in Table 3. For illustration purposes, different configurations and the corresponding migration energies are shown in Figure 7, where the solid red circle represents the migrating molybdenum atom and the dotted circles represent vacancies. Comparing the migration barriers of different configurations and the migration behaviors observed in the TAD simulations, it is shown that although Xe atoms can readily migrate in configuration 1, the migration energy of this mechanism is relatively high (2.49 eV); the reverse migration barriers of configurations 3 and 5 are relatively low (0.33 eV and 0.22 eV), so it is difficult for vacancies to jump out of the Xe vacancy clusters for these three cases. For configurations 2 and 4, the vacancies can separate from the Xe vacancy clusters and diffuse in the matrix. When the vacancies diffuse near the clusters, they easily get absorbed, thus causing the migration of Xe atoms. Therefore, it is considered that the diffusion of Xe atoms is coordinated with the diffusion of vacancies. One of the three vacancies diffuses in the matrix and is absorbed when it diffuses near the cluster, thus causing the Xe atom to diffuse.

### 3.3. The Nucleation of Xe Bubbles in Mo

MD was used to simulate the nucleation process of Xe bubbles. Due to the limited time scale of MD simulation and the high diffusion barrier of Xe atoms in molybdenum, simulations at low temperatures are deemed not feasible. As a result, an attempt to accelerate the Xe bubble nucleation process was made by raising the temperature artificially.

**Figure 8:**
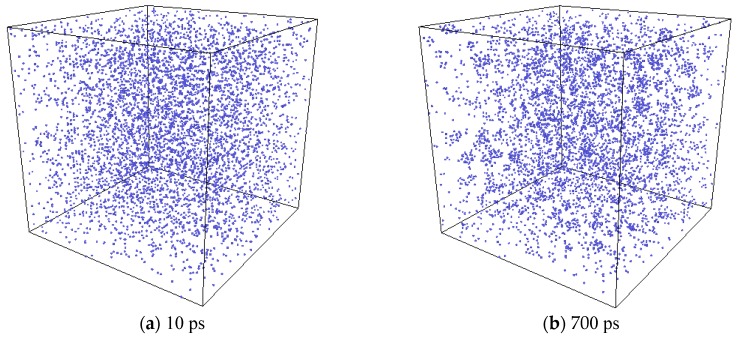

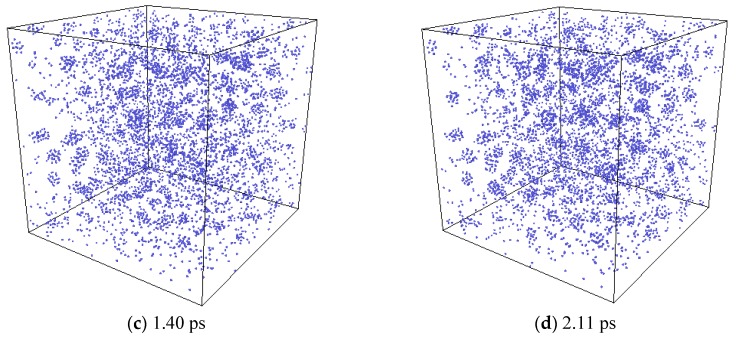
System evolution with a Xe concentration of 2% at a temperature of 2500 K (The blue circle represents xenon atoms.).

**Figure 9:**
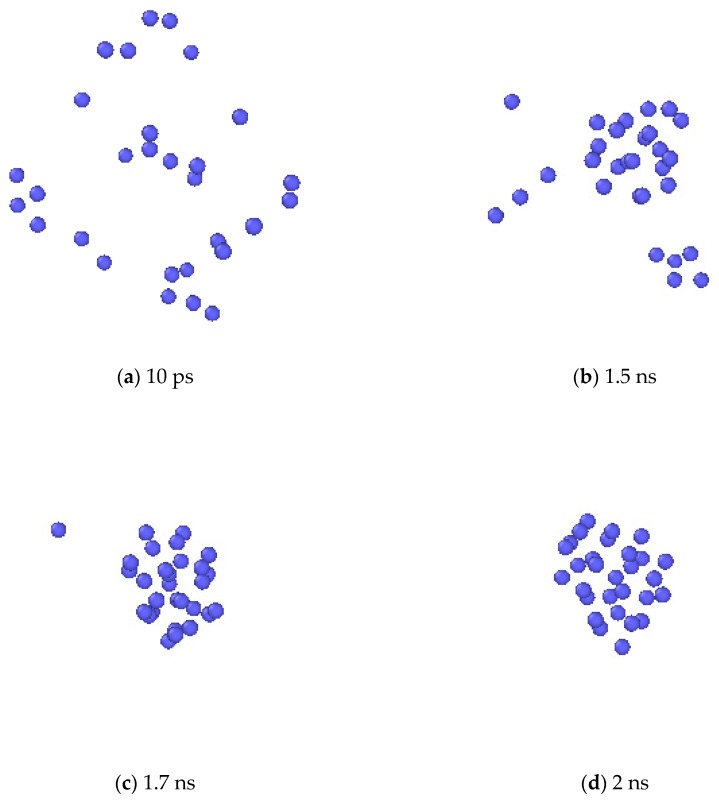
The bubble nucleation process in a close-up view.

Formation of Xe clusters was observed in the simulation. In Figure 8, the evolution of Xe atoms in the system is depicted by the OVITO (the Open Visualization Tool) [33] program. At first, Xe atoms were randomly distributed in the system. At the elevated temperature, small Xe clusters were seen to form. Then the small clusters gradually grew larger by absorbing additional Xe atoms, and finally spherical bubbles are formed. For a clearer illustration, one Xe cluster at the end of the simulation was selected, and the collective evolution behaviors of the Xe atoms that eventually form this cluster are depicted as shown in Figure 9. The process of the Xe cluster formation by the aggregation of scattered Xe atoms can be clearly observed from this figure.

**Figure 10:**
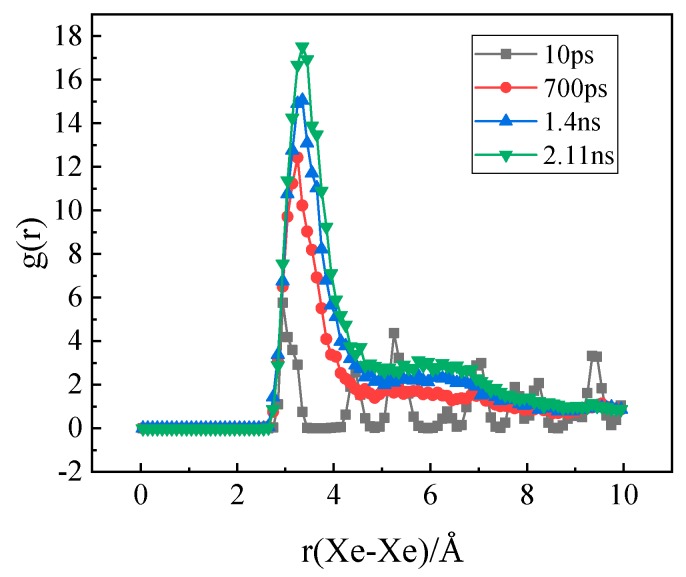
The evolution of the radial distribution function of Xe atoms over time.

At the same time, the radial distribution functions (RDF) of Xe atoms at different times were obtained, as shown in Figure 10. It can be seen that there is only one peak of Xe atoms at the beginning, which indicates a uniform distribution of Xe atoms in the matrix. With the increase of time, the first peak grows gradually and a second peak appears indicating the formation of Xe clusters and the appearance of the second layer atoms in the clusters.

**Figure 11:**
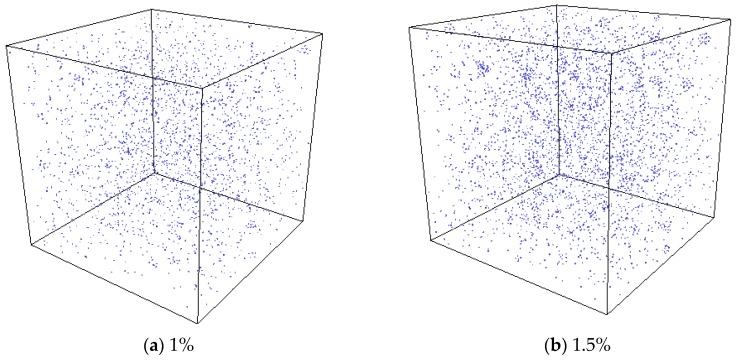

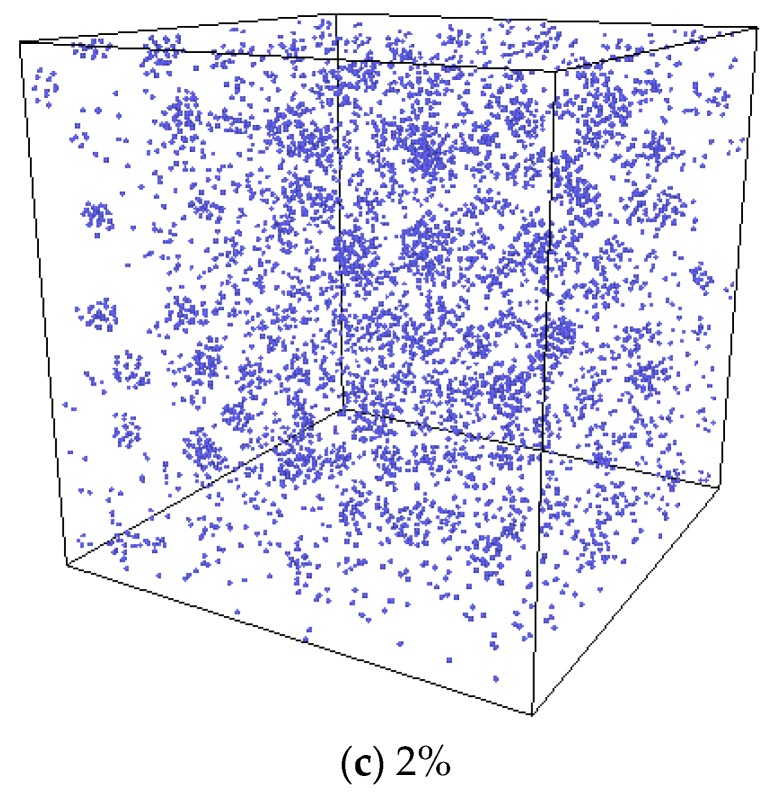
Equilibrium system with different Xe atom concentrations at a temperature of 2500 K.

**Figure 12:**
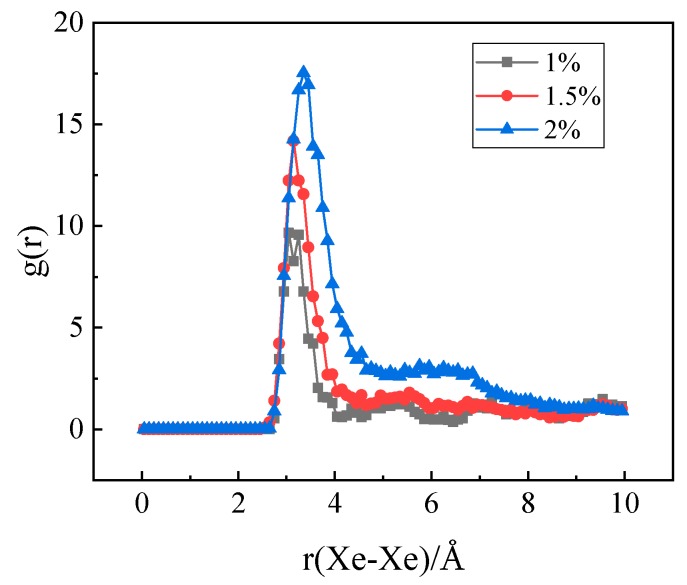
The radial distribution function at different Xe atom concentrations.

In the experiment conducted by Di Yun et al. [20], Xe bubbles were observed when a threshold Xe concentration value was exceeded. It is therefore believed that there could be a threshold value for the Xe concentration beyond which Xe bubbles would form. In order to investigate the effect of Xe concentration on nucleation, MD simulations with different concentrations of substitutional Xe atoms were conducted. The simulated Xe concentrations were 1%, 1.5%, and 2%. Figure 11 shows the distribution of Xe atoms in the as-equilibrated systems at the three different Xe concentrations. It is found that when the concentration of Xe atoms reached 1.5%, the formation of Xe clusters is clearly observed, while when the Xe concentration is 1%, Xe atoms are distributed randomly in the matrix. The RDF of Xe atoms at different concentrations after equilibrium is shown in Figure 12. It is also evident from this figure that nucleation occurred when the Xe concentration reached 1.5%.

### 3.4. The Influence of Xe Bubbles on the Surrounding Vacancies

Xe bubbles can grow up by absorbing vacancies and additional Xe atoms in the matrix. In order to study the interaction between Xe bubbles and the nearby vacancies, the migration energy of a vacancy around Xe bubbles was calculated via NEB.

In order to investigate the influence of bubble size and gas atomic density on the effective influence range of bubbles, two different sized bubbles and four different Xe atomic densities were simulated in this paper. The simulation cases are tabulated in Table 4.

**Figure 13:**
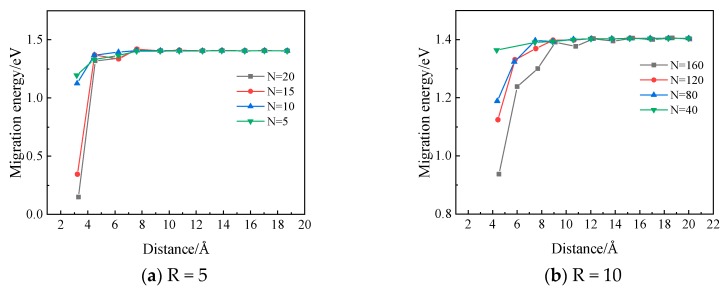
The migration energy of a vacancy around a Xe bubble as a function of the distance to the bubble surface (R is the radius of the bubble in angstroms and N is the number of Xe atoms in the bubble.).

In Figure 13, the migration energy of a vacancy along a <111> direction towards the Xe bubble is demonstrated as a function of the distance between the bubble surface and the vacancy. It can be observed that the migration energy decreases sharply when the distance becomes less than a certain value. This suggests that there is a region around the Xe bubble where the migration behaviors of vacancies can be influenced by the nearby Xe bubble. This is also consistent with the assumption usually taken that there exists an effective capture radius [34] for a defect sink such as a Xe bubble. With the same bubble size, the influence range is observed to increase with increasing number of Xe atoms in the bubble, thus increasing pressure inside the bubble.

## 4. Summary

Static atomistic calculations and MD simulations have been performed in order to investigate the Xe diffusion mechanisms and Xe bubble nucleation and growth behaviors in Mo. From the simulation results, it is concluded that Xe atoms prefer to occupy substitutional sites in Mo. Xe diffuses via vacancy-assisted mechanisms with energy barriers of 1.76 eV, 2.27 eV for Xe combined with two vacancies, and 2.09 eV for Xe combined with three vacancies. The nucleation of Xe bubbles occurs when the concentration of Xe atoms exceeds a threshold concentration value. And this value is ~1.5% for Mo at a temperature of 2500 K. The Xe vacancy cluster is the precursor form of bubbles. With the absorption of Xe atoms and vacancies, Xe bubbles with a spherical shape will gradually form. Meanwhile, the migration energy of a vacancy around a Xe bubble was calculated using the NEB method and this calculation demonstrated that there exists a region around the Xe bubble where the vacancy migration energy is significantly reduced (from 1.4 eV to 0.3 eV for the case where the bubble radius is 5 angstroms and the number of xenon atoms is 15); the influence range increases with increasing number of Xe atoms in the bubble.

## Figures and Tables

**Table 1:** The formation energies of interstitial and substitutional Xe in Mo.

	Octahedral	Tetrahedral	Substitutional Xe+SIA	Substitutional Xe
E_f_(MD)/eV	16.8	16.3	15.3	8.8
E_f_(DFT)/eV [29]	17.1	16.2	15.8	8.8

**Table 2:** Statistical results of different configurations for XeV_2_.

Configuration	Forward Barrier (eV)	Reverse Barrier (eV)
1	1.76	0.64
2	2.27	1.40
3	1.62	0.51

**Table 3:** Migration energy barriers of different configurations for XeV_3_.

Configurations	Forward Barrier (eV)	Reverse Barrier (eV)
1	2.49	2.49
2	2.09	0.79
3	1.73	0.33
4	2.09	1.19
5	1.56	0.22

**Table 4:** Number and density of Xe atoms corresponding to different bubble conditions.

Radius/Angstrom	Density/g·cm^−3^			
	2.08	4.16	6.25	8.33
5	5	10	15	20
10	40	80	120	160

## Supplementary Materials

The following are available online at https://www.mdpi.com/1996-1944/12/15/2354/s1.
